# Characterisation of dissolved organic compounds in hydrothermal fluids by stir bar sorptive extraction - gas chomatography - mass spectrometry. Case study: the Rainbow field (36°N, Mid-Atlantic Ridge)

**DOI:** 10.1186/1467-4866-13-8

**Published:** 2012-11-07

**Authors:** Cecile Konn, Jean-Luc Charlou, Jean-Pierre Donval, Nils G Holm

**Affiliations:** 1 Laboratoire Géochimie Métallogénie, UR Géosciences Marines, Ifremer, Ctr Brest, B.P.70, 29280 Plouzané, France; 2 Department of Geological Sciences, Geochemistry section, Stockholm University, 10691 Stockholm, Sweden

**Keywords:** Sample preparation, Extraction, Organic compounds, Hydrothermal fluids, Ultramafic rocks

## Abstract

The analysis of the dissolved organic fraction of hydrothermal fluids has been considered a real challenge due to sampling difficulties, complexity of the matrix, numerous interferences and the assumed ppb concentration levels. The present study shows, in a qualitative approach, that Stir Bar Sorptive Extraction (SBSE) followed by Thermal Desorption – Gas Chromatography – Mass Spectrometry (TD-GC-MS) is suitable for extraction of small sample volumes and detection of a wide range of volatile and semivolatile organic compounds dissolved in hydrothermal fluids. In a case study, the technique was successfully applied to fluids from the Rainbow ultramafic-hosted hydrothermal field located at 36°14’N on the Mid-Atlantic Ridge (MAR). We show that n-alkanes, mono- and poly- aromatic hydrocarbons as well as fatty acids can be easily identified and their retention times determined. Our results demonstrate the excellent repeatability of the method as well as the possibility of storing stir bars for at least three years without significant changes in the composition of the recovered organic matter. A preliminary comparative investigation of the organic composition of the Rainbow fluids showed the great potential of the method to be used for assessing intrafield variations and carrying out time series studies. All together our results demonstrate that SBSE-TD-GC-MS analyses of hydrothermal fluids will make important contributions to the understanding of geochemical processes, geomicrobiological interactions and formation of mineral deposits.

## Background

Organic geochemistry is of major importance in both geosciences and life sciences [1]. Investigation, identification and quantification of organic compounds (e.g., biomarkers, prebiotic molecules, hydrocarbons) help in understanding the evolution of the Earth and constraining biogeochemical processes that occurred or are still occurring on Earth. The organic geochemistry of rivers, lakes, estuaries, sedimentary basins, terrestrial rocks and oil reservoirs is being extensively studied. Despite the likely significant impact of hydrothermal circulation on the ocean global energy and matter fluxes and the implication of hydrothermal systems in major issues such as the origin of life [2,3], publications on the organic geochemistry of hydrothermal systems are rare. The literature comprises a few studies devoted to the organic contents of hydrothermal sulphide deposits [4], serpentinites [5], carbonate chimneys [6,7] and sediments [8]. In terms of fluids, the abiotic synthesis of dissolved hydrocarbon gases [9-11] as well as the presence of larger dissolved hydrocarbons and other organic molecules [12-14] in fluids from ultramafic-hosted systems at slow spreading ridges has been reported.

Hydrothermal vents are found both on land (e.g. geysers, hot springs) and on the seafloor at Mid-Ocean Ridges (MOR), back arc basins and subduction zones. During hydrothermal circulation, seawater heats up and interacts with rocks in the hot Earth’s crust and mantle. Hydrothermal systems are the places where this modified seawater is expelled as hydrothermal fluids. Water-rock interactions generate gases (CH_4_, H_2_, H_2_S), whereas, major and minor elements such as Fe, Mn, Ca, Li, K, Na, Cl, Si are exchanged between rocks and water. These chemical entities may dissolve in the fluids or precipitate (metal oxide particle). Concentrations in the aqueous phase vary depending on lithologies (rock assemblages), processes that occur during hydrothermal circulation and physico-chemical conditions. For example, major variations in the salinity of the fluids are thought to be due to phase separation [15-18]. Although the inorganic geochemistry of hydrothermal fluids is quite well documented and understood, the study of their organic geochemistry is near its beginning. This is mainly due to sampling and extraction difficulties. Indeed, hydrothermal fluids can be defined as an extremely complex and unusual matrix in which organic compounds are dissolved. The number of samples is limited and collected volumes are small because sampling of hydrothermal fluids is laborious. An extraction method capable of recovering a wide range of organic compounds from small sample volumes of a complex matrix is required and this is a real challenge.

Various sample preparation techniques are available to extract and concentrate analytes from liquids: solid phase extraction (SPE) [19], solid phase microextraction (SPME) [20], membrane extraction with a sorbent interface (MESI) [21], liquid-liquid extraction (LLE) [22], supercritical fluid extraction (SFE) [23], pressurized fluid extraction (PFE) [24] and microwave-assisted solvent extraction [25] to cite a few. The most used techniques for extraction of analytes in liquids are based on LLE or SPE. Over the years, they have proven their efficiency in terms of quantification and most standardised analytical methods use them [19]. However, both techniques involve multiple time-consuming operations. In particular, SPE requires a tedious filtration step to remove suspended particles present in the matrix. The numerous preparation steps associated with SPE extraction multiply sources and risks of contamination. For instance, SPE sorbents and especially polymeric ones constitute major sources of contaminants due to plastics bleeding [26]. Finally LLE uses large amounts of solvents. All these drawbacks cause particular interferences when dealing with small sample volumes containing relatively low amounts (~ppb) of unknown organic compounds dissolved in a complex matrix. Therefore these methods are not the most suitable for the analysis of organic compounds in hydrothermal fluids.

The analysis of volatile and semivolatile organic compounds in aqueous solutions using Stir Bar Sorptive Extraction (SBSE) as the extraction step is gaining acceptance in a wide variety of applications in the environmental (e.g., water analyses), food and biomedical fields [27]. The SBSE is a robust, efficient and convenient technology. It requires minimal sample volumes, the recovery rate is higher than 90% for most nonpolar compounds and 100% of the organic matter sorbed on a stir bar is analysed. The few contaminants associated with SBSE are methylcyclosiloxanes and readily identifiable. The method allows gas chromatographic analysis of organic compounds in aqueous matrices faster than with conventional techniques, omitting time-costly preparation steps and solvents. In addition, the technique has shown great potential to extract organic compounds even from complex matrices (e.g., waste waters, beverages, biological fluids) and to achieve exceedingly low detection limits, under optimised conditions, by being times more sensitive than direct SPME [28,29]. For example, Ochiai and Nakamura [30] measured sub-part per trillion (sub-ppt) levels of off-flavor compounds in drinking water. In seawater, polyaromatic hydrocarbons (PAHs) have been detected down to the sub-ppt levels [31] and Pérez-Carrera et al. [32] reported limits of detection (LOD) of the order of the ppt for PCBs. León et al. [33] obtained LOD in the range of 0.04 to 11 ppt for semivolatile organic compounds (2 < LogK_o/w_ < 7.66, see explanation in the next paragraph) in salted tap water. Nevertheless, SBSE, like any other analytical method, has some limitations for extraction of certain compounds and the attainment of such low LOD may require further sample preparation, e.g., pH adjustment, back extraction or derivatisation [34].

As mentioned in a review on SBSE theory and applications [27], SBSE is by nature an equilibrium technique based on the partitioning of solutes between a polymer phase (PolyDiMethylSiloxane (PDMS)) and the aqueous matrix. This polymer is in a liquid-like state at room temperature, resulting in the retention of the analytes by dissolution into the bulk of the PDMS (this phenomenon is called sorption) rather than by adsorption on a surface which is the retention process involved in other extraction techniques. The partition equilibrium is correlated with the octanol-water partition coefficient (K_o/w_) which can be defined by equation (1):

(1) $$K_{O/W}\approx K_{\mathrm{PDMS}/W}=C_{\mathrm{SBSE}}/C_{W}=\left(m_{\mathrm{SBSE}}/m_{W}\right)\times \left(V_{W}/V_{\mathrm{SBSE}}\right)$$

where C_SBSE_ and C_W_ are the analyte concentration in the SBSE and the water phase, respectively, m_SBSE_ and m_W_ are the mass of analyte in the SBSE and the water phase, respectively and V_SBSE_ and V_W_ are the volume of PDMS and water phase, respectively.

The retention and the recovery rate of a molecule mainly depend on K_o/w_ and on the sample to PDMS volumes ratio (β), even though concentration of analytes, pH, polarity of the matrix and extraction time have some effect [27,35]. To a lesser extent, analytical parameters such as desorption flow, CIS initial temperature and CIS splitless time may also affect the retention and recovery rate of a compound [36]. Using β, equation (1) can be restated as:

(2) $$K_{O/W}/\beta =m_{\mathrm{SBSE}}/m_{W}=m_{\mathrm{SBSE}}/\left(m_{0}-m_{\mathrm{SBSE}}\right)$$

where m_0_ is the total amount of analyte originally present in the water sample. For a more detailed theoretical study, we urge the reader to refer to a paper by Baltussen et al. [28] who have been pioneers in the SBSE method development.

**Figure 1:**
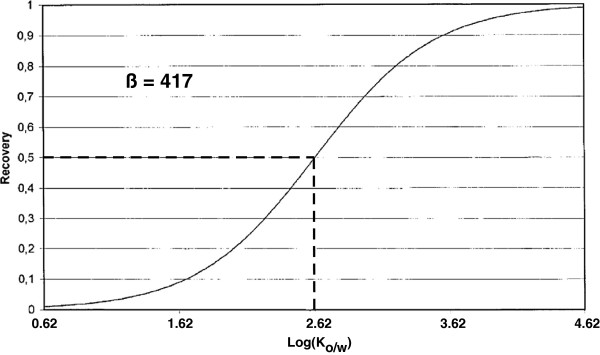
**Modified after Baltussen et al. **[28]. Recovery as a function of the octanol-water partitioning constant log(K_o/w_).

In our case β = 417 (V_W_ = 10 mL and V_SBSE_ = 24 μL) and this corresponds to a recovery > 50% for compounds with a K_o/w_ > 2.62 in pure water (Figure 1). However, the partition coefficients may vary with the pH; typically for polar compounds [37]. Also, the presence of dissolved salts and gases, as well as particulates to which organic molecules can bind, are likely to affect the way compounds partition between PDMS and hydrothermal fluids. As such, the recovery rate in hydrothermal fluids may deviate somewhat from that in pure water. Nevertheless, we provide in Table 1 an estimate of the range of log(K_o/w_) values in pure water for the type of compounds that are very likely inherent to hydrothermal fluids*.* In this study, we show that SBSE – Thermal Desorption – Gas Chromatography – Mass Spectrometry (TD-GC-MS) is an effective and reliable method capable of isolating a wide range of organic molecules from small sample volumes of the complex matrix that are hydrothermal fluids. This technique is therefore suitable and essential for the study of the organic geochemistry of hydrothermal fluids. We demonstrate this by applying SBSE to the analyses of fluids from the Rainbow ultramafic-hosted hydrothermal field (36°14’N, Mid-Atlantic Ridge).

**Table 1:** Summary of the groups of compounds detected (+) and not detected (nd) in fluids from the Rainbow hydrothermal field over 3 years and in the deep seawater extract

***Compound***	***Log(K***_***o/w***_***)***	***2005***	***2007***	***2008***	***Deep seawater***
n-alkanes (C_9-19_)	5.5-8.2	C_9-14_	C_11_	C_10_	C_11_
branched saturated alkanes (C_9-12_)	5.0-6.8	+	nd	+	nd
cycloalkanes (C_9-11_)	4.9-6.0	+	nd	nd	one C_9_
phenol	1.5	+	nd	+	nd
toluene	2.7	+	+	+	+
ethylbenzene	3.2	+	nd	+	nd
xylene	3.2*	+	nd	+	+
styrene	2.7	+	+	+	+
other alkylated benzenes (C_9-12_)	3.7-5.0	nd	?	?	one C_9_H_12_
naphthalene	3.4	+	+	+	+
methyl and dimethyl naphthalenes	> 3	+	+	+	nd
PAHS (C_12-16_)	4.2-5.2	+	+	+	nd
n-fatty acids (C_8-18_)	3.4-8.2	C_9-18_	C_8-10_	C_9-16_	C_6_ and C_14-16_

* [38].

(?) stands for compounds for which further investigation is needed to confirm their presence. Results were identical for the two samples in 2007 with respect to the compounds listed in this table, therefore it appears only one column for 2007. C_n_ is the carbonated chain length of the identified compounds. Additionally, ranges of Log(K_o/w_) values at 25°C for pure water are given. These values are meant to give a rough estimate only. The reader should bear in mind that the partition between seawater or hydrothermal fluids and PDMS will somewhat differ from these values. Precise identification of isomers was not possible without the use of synthetic standards so that ranges of values have been determined based on log(K_o/w_) values for compounds with the same raw formula. Values were obtained from the SciFinder® data base.

## Experimental

### Features of the study area

**Figure 2:**
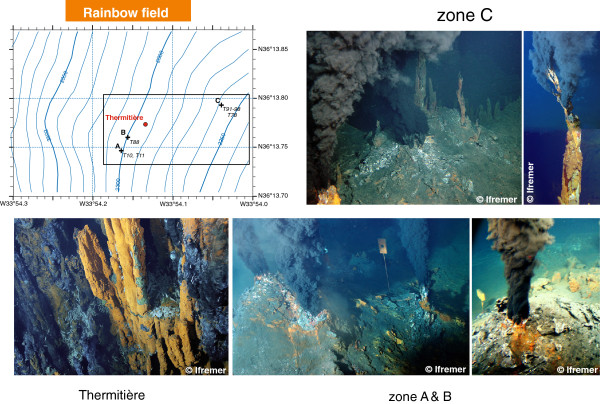
**Left is a picture of a black smoker taken on the Rainbow field during the MoMARDREAM-Naut cruise in 2007 by the Nautile camera.** Right is the bathymetric map of the Rainbow hydrothermal field: modified after Charlou et al. [16]. The map was established during the FLORES cruise, but the smokers were sampled again during the EXOMAR, MoMARDREAM-Naut and MOMAR08-Leg2 cruises. The box roughly marks the boundary of the active zone of the site, which can be described by 3 main active sub-areas: south-west (around **A** and **B**), central (Thermitière, dot) and north-east (around **C**). Sampling locations have been marked by crosses and have been referred in the text to **A**, **B** and **C**. The Twister® # that was used for extraction of the fluid sample stands next to each cross (to be related to Table 2).

The Rainbow site is an unsedimented hydrothermal field located on the Mid-Atlantic Ridge (MAR), south of the Azores, at 36°14’N, 33°54’W and at 2300 m depth [39]. It is located at the intersection of the non-transform fault system and the ridge faults, on the west-facing flank of the Rainbow ridge at the northeastern corner of the south Azores Mid-Atlantic Ridge (AMAR) segment. The field measures is about 250 m (east–west) by 60 m (north–south) and consists of three qualitatively distinct active areas (Figure 2): Thermitière, an organ pipe-like structure with both hot and diffuse fluids that hosts most of the biota, the north-east zone (**C** on Figure 2), which consists of very active short black smokers and the south-west zone (**A, B** on Figure 2), which is less active, with a lot of old chimneys and a few tall candelabrum-like active chimneys. The Rainbow field is located on peridotite-rich mantle outcrops that are associated with emission of large amounts of CH_4_[40]. These peridotites are undergoing serpentinisation, leading to production of a large amount of H_2_[9,16]. The temperature of the fluids is around 360°C leading to phase separation in the subseafloor [39,41]. High chlorinity (780 mmol kg^-1^), low pH (pH = 3–4), high concentrations of metals (e.g. [Fe] = 24 mmol kg^-1^), alkali metals and alkaline earth metals [41], low concentration of dissolved SiO_2_, and high levels of dissolved hydrogen (16 mmol kg^-1^) and methane (2.5 mmol kg^-1^) [16,40] characterise the inorganic geochemistry of the Rainbow fluids. Finally there have been reports that hydrocarbons and oxidized organic compounds are dissolved in the Rainbow fluids [12,13].

### Sample collection and preparation

**Figure 3:**
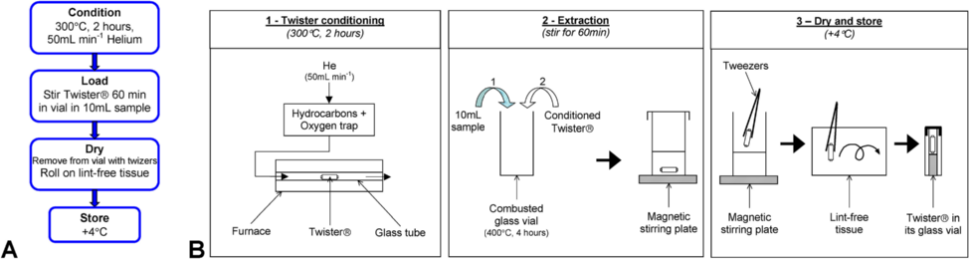
Summary diagram of the SBSE procedure (A) and schematic representation of the sample preparation step (B).

All glassware used was pre-combusted at 400°C for 4 hours to remove any trace of organic matter. The commercial stir bars (Twisters®) used in this study consist of a magnetic rod in a tubular glass housing coated with 24 μL PDMS (length = 10 mm, film thickness = 0.5 mm). They were purchased from Gerstel GmbH & Co. KG (*Mülheim an der Ruhr, Germany*). Twisters® were conditioned prior to use by thermal desorption at 300°C for 2 hours under a purified helium (He) flow (50 mL min^-1^) (Figure 3). For each batch of conditioned Twisters® one was kept as a dry blank reference.

Hot fluids of the Rainbow ultramafic-hosted hydrothermal field and deep seawater were collected in titanium syringes using the same procedure. Sampling was conducted by the ROV Victor 6000 during the EXOMAR (2005) and MOMAR08-Leg2 (2008) [42] cruises and by the Nautile during the MOMARDREAM-Naut (2007) cruise [43]. All cruises were made under the auspices of Ifremer, France. Hot fluid samples were taken as deep as possible within black smokers to minimise seawater mixing. Deep seawater samples were taken in the vicinity of the Rainbow field where hydrothermal input could be discounted. Table 2 lists the samples used in this study and gives their general characteristics, whereas Figure 2 shows the distribution of the sampling locations over the Rainbow hydrothermal field. Only an aliquot of the total sample volume could be dedicated to organic geochemistry. As soon as the syringes were recovered, 10 mL aliquots of fluid samples were accurately measured using a pipette and transferred from the titanium syringes into glass vials where the conditioned Twisters® were added and allowed to stir for 60 min at 300 rpm. Twisters® were then removed, dried on lint-free tissue and stored in their airtight glass vials at +4°C until analysis by TD-GC-MS. A summary of these operations is shown schematically in Figure 3.

**Table 2:** General features of the samples used in this work

***Year***	***Location***	***Sample name***	***Twister® #***	***Depth(m)***	***pH***	***T(°C)***	***H***_***2***_***S(mM)***	***Cl***^***-***^***(mM)***
2005	A	EXO-D6-Ti1	T10 & T11	2306	3.79	353	0.963	774
2007	B	MAD-D8-Ti1D	T88	2305	3.36	350	-	754
2007	C	MAD-D6-Ti2G	T78	2265	3.23	353	-	761
2008	C	MOM-D4-Ti3	T91-T98	2258	3.18	360	-	716
2005-8	-	Deep seawater	-	2230-90	7.84	2	<0.1	547

For location refer to Figure 2.

Typical concentrations in deep seawater are given for reference. A deep seawater sample was collected each year outside of the active zone (outside the box on Figure 2) and the values are averages of those 3 samples.

### Synthetic standards

We chose for this preliminary study to focus on the groups of compounds that were the most relevant to hydrothermal organic geochemistry: n-alkanes, linear fatty acid and aromatic hydrocarbons [13,44]. Other aliphatic hydrocarbons (branched and cyclic) have been reported in hydrothermal fluids and would also be of interest, but compounds of these homologous series have mass spectra that are too similar to be accurately identified without the use of standards of individual compounds. Individual custom standards are very expensive. Moreover these additional hydrocarbons were not essential to show the suitability of SBSE-TD-GC-MS for the analysis of hydrothermal fluids. Therefore we considered such a purchase unnecessary.

Custom mixtures were purchased from *LGC standards SARL, Molsheim, France*: C_8_-C_20_ linear fatty acids in isooctane at 1 mg/mL; C_9_-C_20_ n-alkanes in MeOH; monoaromatic hydrocarbons (BTEX) in MeOH at 200 μg/mL; PAHs in MeOH at 1 mg/mL. Three separate solutions were prepared by spiking MQ water (18.2 MΩ) with fatty acids (50 μg/L); n-alcanes (10 μg/L); PAHs and BTEX (10 μg/L). Extraction was performed using to the same procedure as for the hydrothermal fluids (see § “Sample collection and preparation”).

### Instrumentation and analytical conditions

Analyses of the stir bars were performed by TD-GC-MS. The Twisters® were thermally desorbed in the thermal desorption system (TDS-2, *GERSTEL GmbH & Co. KG, Mülheim an der Ruhr, Germany*) mounted on a 6890 Agilent GC (*Agilent Technologies, Little Falls, DE, USA*) equipped with a 5973 quadrupole mass spectrometer detector (MSD). The TDS was coupled to a cooled injection system (CIS4, *GERSTEL GmbH & Co. KG, Mülheim an der Ruhr, Germany*) for cryofocusing the analytes prior to their transfer onto the column. Liquid nitrogen was used to cool and maintain the CIS at −100°C while the Twister® was desorbed in the TDS in the splitless mode at 300°C for 5 min under He flow. The CIS was then heated to 250°C. Separation was achieved on an HP5-MS (*Agilent Technologies, Little Falls, DE, USA*) capillary column (30 m × 0.25 mm i.d. × 0.25 μm film thickness). The GC column temperature was held first at 40°C for 1 min, then ramped from 40 to 320°C at 12°C min^-1^ and held at 320°C for 2 min. Helium was used as carrier gas with a flow of 1.2 mL min^-1^. The mass spectrometer was operated simultaneously in full scan and selected-ion monitoring (SIM) modes for the analysis of standard solutions, whereas hydrothermal fluids samples could only be analysed in full scan mode. Electron ionisation mass spectra were recorded in the 10 to 500 amu range at 70 eV ionisation energy. The dwell time was adjusted in the SIM mode to obtain 2 to 3 cycles / sec. Data were acquired and processed by the Chemstation software. Retention times (Rt) of the targeted compounds were determined with the help of the NIST08 library, using both the full scan and SIM chromatograms of the standard solutions. Unequivocal identification of individual organic compounds in hydrothermal fluids was possible using both the Rt of the standards and extracted ion chromatograms.

## Results and discussion

### Identification of compounds in hydrothermal fluids using standard mixtures

Konn et al. [13], successfully identified organic compounds in several hydrothermal fluid samples based on comparison with the NIST02 library associated with consistent Rt. The present study confirms these early pioneering results using standard mixtures for n-carboxylic acids, n-alkanes as well as mono- and poly- aromatic hydrocarbons. Simultaneous analyses of standard solution extracts in full scan and SIM mode enabled accurate determination of the retention times (Rt) of the targeted compounds. Retention times have been proposed for these compounds in hydrothermal fluids based on previous studies [13,44] and the NIST08 library (this study). Retention times values were generally very similar in standard solutions and hydrothermal fluids, which confirms that our previous peak assignment was correct. Chromatograms of hydrothermal fluids usually differed from those of the standard solutions. These variations are unlikely to be artifacts due to matrix differences because the extraction rate of compounds of a homologous series should be affected in the same way. Our results suggested that the distribution patterns of each group of compounds in hydrothermal fluids are specific. Such patterns are likely the result of the particular geochemical processes controlling the formation and dissolution of organic compounds in hydrothermal fluids.

#### n-Carboxylic acids

**Figure 4:**
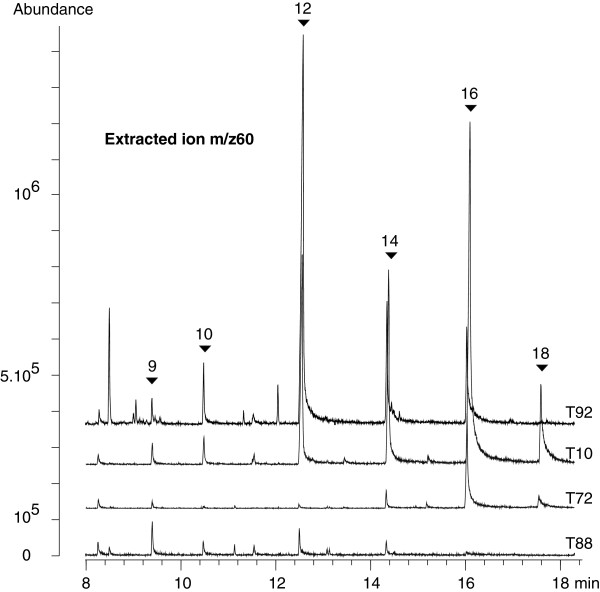
**Extracted ion chromatograms at the qualifier ions *m*/*z *60 of the SBSE–TD-GC–MS analysis of 3 hydrothermal fluid samples (T10, T88, T92) and a deep seawater sample (T72).** Top to bottom: T92, T10, T72 and T88. Description of these samples is found in Table 2. n-Carboxylic acids peaks are pointed out (full triangle) with their respective carbon number standing above. Rt are given in Table 3.

Peaks of underivatized fatty acid have a tendency to tail on a HP5-MS column due to the polarity of the compounds (Figure 4). Therefore peaks may be easily detected by the naked eye. However, detection of n-fatty acid peaks was best achieved by targeting ion m/z 60 (major) and 73 (confirmation) in the standard solution and by extracting ion m/z 60 in hydrothermal fluid samples. This major ion is characteristic of carboxylic acids and forms via a McLafferty rearrangement [45]. The whole series of n-fatty acids (C_8:0_-C_18:0_) was detected in the standard mixture. The best response was obtained for C_9:0_-C_16:0_ compounds, whereas C_8:0_ and C_17:0_-C_19:0_ had a much higher LOD. This can be explained by the low K_o/w_ value of shorter chain fatty acids and the weak volatility of longer chain fatty acids when underivatized. We observed a slight offset between the Rt of the standards and those of the hydrothermal fluids. Carboxylic acids in the range of C_8:0_ to C_14:0_ eluted 0.02-0.09 min earlier in the hydrothermal fluids whereas the C_16:0_-C_18:0_ eluted 0.06-0.1 min later (Table 3). We believe that this off-set is due to concentration differences because the higher the concentration the larger were the peaks and the longer their tails. This resulted in a shift in Rt [13].

**Table 3:** **Retention times (Rt**_**stds**_**) of C**_**8:0**_**-C**_**20:0**_**carboxylic acids obtained by SBSE-TD-GC-MS analyses of a standard mixture (50 μg/L)**

***Compounds***	***Molecular formula***	***Rt***_***stds***_***(min)***	***Rt***_***fluids***_***(min)***
octanoic acid	C8H16O2	8.30	8.24†
nonanoic acid	C9H18O2	9.47	9.43*
decanoic acid	C10H20O2	10.61	10.52*
undecanoic acid	C11H22O2	11.68	-
dodecanoic acid	C12H24O2	12.67	12.58*
tridecanoic acid	C13H26O2	13.59	-
tetradecanoic acid	C14H28O2	14.44	14.41*
pentadecanoic acid	C15H30O2	15.24	15.23†
hexadecanoic acid	C16H32O2	16.02	16.12*
heptadecanoic acid	C17H34O2	16.78	-
octadecanoic acid	C18H36O2	17.65	17.61*
nonadecanoic acid	C19H38O2	18.27	-
eicosanoic acid	C20H40O2	-	-

Detection of the compounds was achieved using ions m/z 60 and 73. The NIST08 library was used for identification. Retention times (Rt _fluids_) obtained in hydrothermal fluids are given for direct comparison: * This study, † [13].

Linear carboxylic acids detected in the hydrothermal fluids were in the C_9:0_-C_18:0_ range and showed an even carbon number predominance. This may indicate a biogenic contribution as living organisms are preferentially made of even carbon numbered fatty acids in the C_12:0_-C_22:0_ range [46]. By contrast, Fischer Tropsch Type abiogenic reactions, that likely occur in hydrothermal systems, generate C_6:0_-C_22:0_ n-fatty acids without carbon number selectivity, but result in a far larger number of shorter chain compounds (C_6:0_-C_10:0_) [47,48]. Under hydrothermal conditions and at temperatures above 250°C, cracking processes might also be responsible for the presence of short chain fatty acids in hydrothermal fluids from the Rainbow field [48]. The distribution of fatty acids in hydrothermal fluids may well be affected by dissolution, adsorption and complexation reactions that likely occur during hydrothermal circulation. It is therefore premature to draw conclusions at this stage based on the present preliminary observations.

#### n-Alkanes

**Figure 5:**
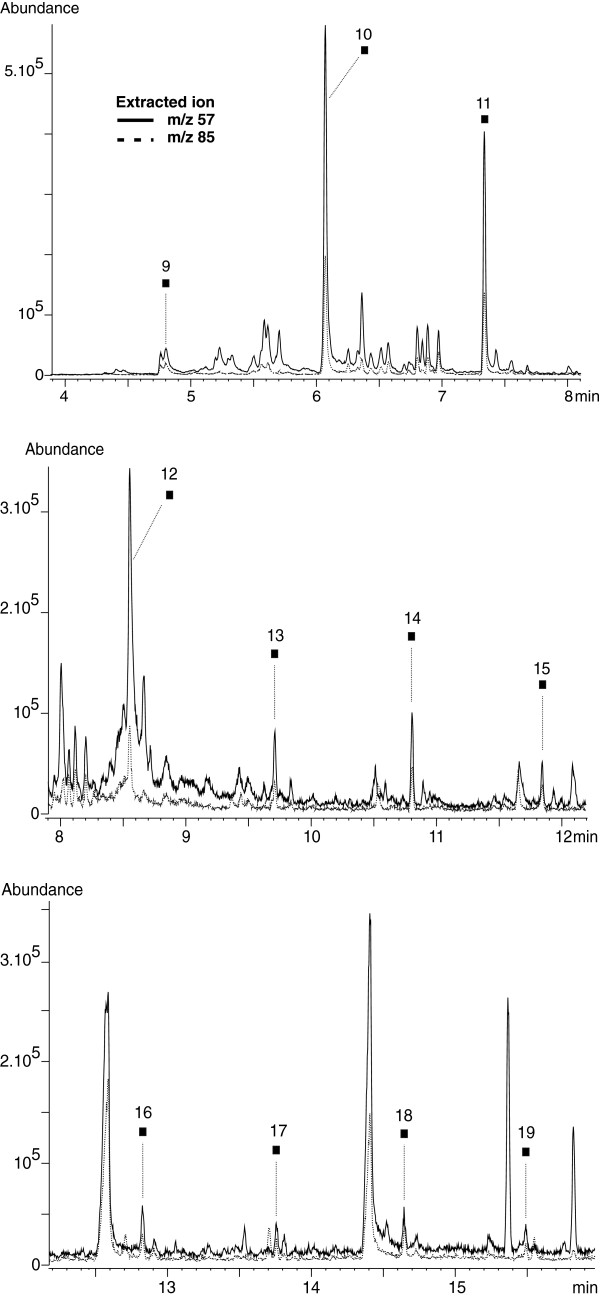
**Extracted ion chromatograms at the qualifier ions *m/z *57 (full line) and *m/z *85 (dashed line) of the SBSE-TD-GC-MS of the 2005 hydrothermal fluid extract (Table**2**).** n-Alkanes were only detected that year. The corresponding peaks are pointed out with their respective carbon number standing above. Rt are given in Table 4.

We recorded a signal for each of the n-alkanes present in the standard solution. The highest response was obtained for C_11_ and C_12_ n-alkanes. The peak’s areas corresponding to C_9_, C_10_ and C_13_ were about 50% those of the C_11_ and C_12_ n-alkanes. We observed a consistent decrease of the peak areas from C_11_ to C_16_ (100% to 3.5%). C_15_-C_20_ were easily detected, albeit exhibiting a much weaker signal (~1.5% of C_11_ peak area). n-Alkanes were clearly identified in the Total Ion Currents (TICs) of hydrothermal fluids by extracting ion m/z 57 (major ion) and 85 (confirmation ion). These ions are characteristic of n-alkanes and are formed via simple fragmentation mechanisms with bond cleavage. The whole series of n-alkanes except for eicosane could be detected in the hydrothermal fluid extracts (Figure 5). The Rt obtained for C_9_-C_19_ n-alkanes in hydrothermal fluids of the Rainbow site were almost identical (+ 0.01-0.06 s) to the synthetic standards ones (Table 4). C_16_-C_19_ responses were low and of the same order of magnitude as observed for the standard solution. The highest response was obtained for decane (C_10_) and was about 25% higher than for undecane (C_11_) and dodecane (C_12_). Also, the C_13_ signal was far lower than that of C_14_ contrary to what was observed in the standard solution. These variations in the distribution of carbon species may be indicators of specific chemical processes. The absence of C_20_ is probably due to a too high LOD. C_12_ and C_15_ were not reported previously because they were masked on the TICs by much more abundant co-eluting compounds. They could only be detected in this work by extracting ion m/z 57 and 85.

**Table 4:** **Retention times (Rt**_**stds**_**) of C**_**9**_**-C**_**20**_**n-alkanes obtained by SBSE-TD-GC-MS analyses of a standard mixture (10 μg/L)**

***Compounds***	***Molecular formula***	***Rt***_***stds***_***(min)***	***Rt***_***fluids***_***(min)***
nonane	C9H20	4.75	4.76
decane	C10H22	6.01	6.05
undecane	C11H24	7.28	7.32
dodecane	C12H26	8.49	8.52
tridecane	C13H28	9.65	9.70
tetradecane	C14H30	10.74	10.79
pentadecane	C15H32	11.77	11.81
hexadecane	C16H34	12.77	12.81
heptadecane	C14H36	13.69	13.74
octadecane	C18H38	14.57	14.62
nonadecane	C19H40	15.41	15.47
eicosane	C20H42	16.21	-

Detection of the compounds was achieved using ions m/z 57 and 85. The NIST08 library was used for identification. Retention times (Rt _fluids_) obtained in hydrothermal fluids samples of this study are given for direct comparison.

#### Monoaromatic hydrocarbons

**Figure 6:**
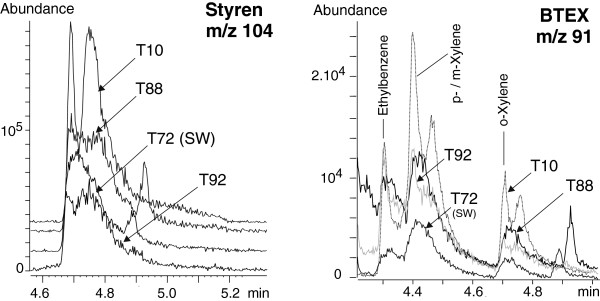
**Zooms of extracted ion chromatograms at the qualifier ion *m/z *104 (left) and *m/z *91 (right) of the SBSE-TD-GC-MS analysis of 3 hydrothermal fluid samples (T10, T88, T92) and a deep seawater sample (T72).** Arrows point out their respective traces. On the right panel, T92 and T10 signals are represented in dashed line while T72 and T88 appear in full line. The styrene peak elutes at 4.71 min (left) and the other BTEX in the 4.31 -4.71 min range (right). Refer to Table 2 and Table 5 for description of the samples and Rt values.

**Figure 7:**
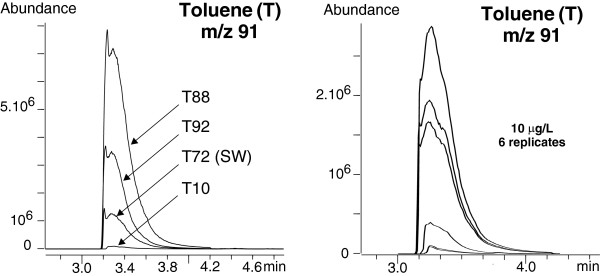
**Zooms of extracted ion chromatograms at the qualifier ion m/z 91 of the SBSE-TD-GC-MS analysis of 3 hydrothermal fluid samples (T10, T88, T92) and a deep seawater sample (T72) (left panel); and 6 replicates of MQ water spiked with toluene at 10 μg/L (right panel).** On the left panel, arrows point out the respective traces of natural samples. Refer to Table 2 and Table 5 for description of the samples and Rt values.

In our standard solution, monoaromatic hydrocarbons were much easier targeted in the SIM mode, using m/z 91, 103.9, 104.9, 120 and 133.9, than in the full scan mode because of co-elution. Co-elution occurred because of the relatively high initial temperature (40°C) of the GC oven, which could not be lowered. The tropylium ion (m/z 91) is characteristic of BTEX but does not enable identification. The use of other confirmation ions and the NIST08 reference spectra were necessary for discrimination between isomers. All monoaromatic hydrocarbons were successfully identified in the standard mixture. Only toluene, styrene, p-, m-, o-xylene and ethylbenzene were detected in the hydrothermal fluids (Figure 6, Figure 7). The Rt obtained for the standard analysis and for hydrothermal fluids correlated very well (Table 5). The toluene response was highly variable in the hydrothermal fluids (Figure 7). This was also the case for the standard solutions and is commonly observed in SBSE-TD-GC-MS because of the high volatility of toluene (J. Guyomarch, personal communication). We do not exclude the occurrence of other BTEX but probably at concentrations below current LOD. Therefore the Rt for all compounds of the standard solution were reported in Table 5.

**Table 5:** **Retention times (Rt**_**stds**_**) of monoaromatic hydrocarbons obtained by SBSE-TD-GC-MS analyses of a standard mixture (BTEX at 10 μg/L)**

***Compounds***	***Molecular formula***	***Rt***_***stds***_***(min)***	***Rt***_***fluids***_***(min)***
toluene (**T**)	C7H8	3.28	3.26
ethylbenzene	C8H10	4.33	4.34
p-xylene / m-xylene	C8H10	4.45	4.43
o-xylene	C8H10	4.74	4.71
styrene	C8H8	4.74	4.71
isopropylbenzene	C9H12	5.10	-
n-propylbenzene	C9H12	5.49	-
1,3,4-trimethylbenzene	C9H12	5.61	-
tert-butylbenzene	C10H14	5.96	-
1,3,5-trimethylbenzene	C9H12	6.00	-
sec-butylbenzene	C10H14	6.21	-
paraisopropyltoluene	C10H14	6.39	-
n-butylbenzene	C10H14	6.81	-

Detection of the compounds was achieved using ions m/z 91, 103.9, 104.9, 120 and 133.9. The NIST08 library was used for identification. Retention times (Rt _fluids_) obtained in hydrothermal fluids samples of this study are given for direct comparison.

#### Polyaromatic hydrocarbons (PAHs)

**Figure 8:**
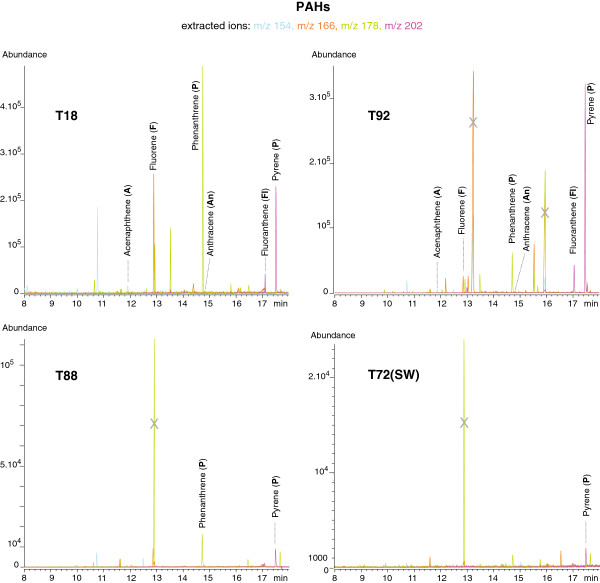
**Extracted ion chromatograms at the qualifier ions m/z 154 (blue), m/z 166 (orange), m/z 178 (green), m/z 202 (purple) of the SBSE-TD-GC-MS analysis of 3 hydrothermal fluid samples (T10, T88, T92) and a deep seawater sample (T72).** Qualifier ions correspond to the parent ions of PAHs that are pointed out on the chromatograms. Refer to Table 2 and Table 6 for description of the samples and Rt values.

**Figure 9:**
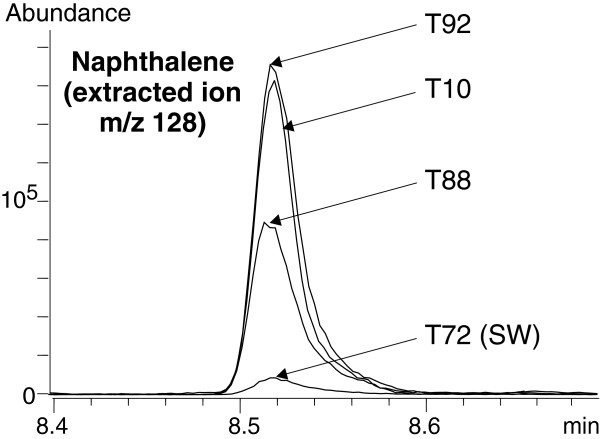
**Zoom of extracted ion chromatograms at the qualifier ion m/z 128 (naphthalene) of the SBSE-TD-GC-MS analysis of 3 hydrothermal fluid samples (T10, T88, T92) and a deep seawater sample (T72).** Arrows point out each sample signal. See Table 2 and Table 6 for description of the samples and Rt values.

PAHs were clearly evident on both full scan and SIM chromatograms of the standard solution. The resolution was extremely good as was the peak shape (Figure 8). The signal obtained for acenaphthene and fluorene was twice as low, whereas the response for other PAHs relative to napthlalene was 65-70%. Detection of the whole series of PAHs in hydrothermal fluid samples was possible and achieved by extracting the parent ions (Table 6). Extracted ion chromatograms for PAHs are presented in Figure 8. Naphthalene showed the highest response and is thus presented separately for scale reasons (Figure 9). The Rt values were almost identical in the standard solutions and the natural samples (Table 6). Phenanthrene and pyrene signals were more than one order of magnitude higher than their respective isomers, anthracene and fluoranthene, in hydrothermal fluids. Unlike their behaviour in the standard solution, they all exhibited a similar response. The reason why anthracene and fluoranthene seem to occur in very low amounts and whether it is significant in terms of geochemical processes will be worth investigating.

**Table 6:** **Retention times (Rt**_**stds**_**) of polyaromatic hydrocarbons (PAHs) obtained by SBSE-TD-GC-MS analyses of a standard mixture (10 μg/L)**

***Compounds***	***Molecular formula***	***Rt***_***stds***_***(min)***	***Rt***_***fluids***_***(min)***	***m/z***
naphthalene (N)	C10H8	8.47	8.52	128
acenaphthene (A)	C12H10	11.86	11.87	154
fluorene (F)	C13H10	12.85	12.86	166
phenanthrene (P)	C14H10	14.70	14.71	178
anthracene (An)	C14H10	14.79	14.81	178
fluoranthene (Fl)	C16H10	17.04	17.06	202
pyrene (P)	C16H10	17.46	17.47	202

Detection of the compounds was achieved using the molecular ion for each compound (last column). The NIST08 library was used for identification. Retention times (Rt _fluids_) obtained in hydrothermal fluid samples of this study are given for direct comparison.

### Blank and control experiments

**Figure 10:**
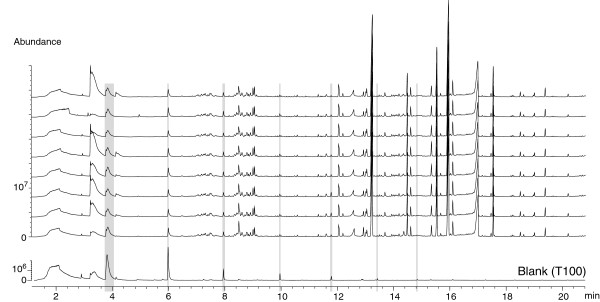
**(A) is an overlay of TD-GC-MS TIC traces of SBSE extracts of 8 aliquots of the same fluid sample from the Rainbow hydrothermal vent field in 2008 (refer to Table**2**and Figure**2**).** Offset of the traces was enabled for clarity. Seven stir bars, T91 to T97, were analysed on the same day; the last one, T98 (top trace) was analysed one day after. Peaks highlighted in grey are the Twister® characteristic peaks. The bottom trace was obtained for the dry blank experiment T100 (refer to § Sample collection and preparation and § Blank and control experiments) and is characteristic to a clean conditioned Twister® (2 h, 300°C, He 50 mL min^-1^). Note that the scale for the latter is different.

Because contamination cannot be totally excluded when dealing with natural samples, we put considerable effort into identifying contaminants. Dry blank experiments were carried out routinely. The dry blank Twister® was not used for sample extraction but was stored together with the rest of the batch, ensuring the detection of any contamination that could have occurred during conditioning, storage or anything that was not related to sample preparation. TD-GC-MS analyses of these dry blank Twisters® enabled us to establish that conditioning and storage were not sources of contamination in any of the samples used in this work. One representative example (T100) of the TIC of a dry blank can be seen in Figure 10. The TIC of a cleaned Twisters® is very characteristic, and consists of a series of siloxane peaks that have been highlighted in grey on all the TICs of this paper. The nature and Rt of these siloxanes are given in Table 7. These peaks always occur and cannot be removed by further conditioning.

**Table 7:** Name, molecular formula and retention times (Rt) of the characteristic compounds that are leaked from the PDMS phase of the Twisters®

***Compounds***	***Molecular formula***	***Rt (min)***
cyclotrisiloxane, hexamethyl-	C6H18O3Si3	3.80
cyclotetrasiloxane, octamethyl-	C8H24O4Si4	6.03
cyclopentasiloxane, decamethyl-	C10H30O5Si5	7.98
cyclohexasiloxane, dodecamethyl-	C12H36O6Si6	9.99
cycloheptasiloxane, tetradecamethyl-	C14H42O7Si7	11.82
cyclononasiloxane, octadecamethyl-	C18H54O9Si9	14.88

**Figure 11:**
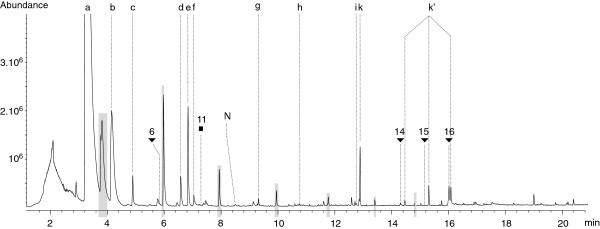
**Representative TIC trace of the SBSE-TD-GC-MS analysis of 2000 m deep seawater in the neighbourhood of the Rainbow hydrothermal field.** This particular sample was collected in 2007 at a depth of 2291 m. Major contaminants are: (**a**) toluene; (**b**) benzene; chloro-; (**c**) oxime, methoxyphenyl; (**d**) benzene, 1,x-dichloro; (**e**) octane, 1-chloro; (**f**) siloxane; (**g**) decane, 1-chloro; (**h**) naphthalene, 2-chloro; (**i**) siloxane; (**k**) diethylphthalate; (k’) phthalates. Peaks highlighted in grey are the Twister® characteristic peaks. Numbers stand for the carbonated chain lenght of n-alkanes (full squares) and n-carboxylic acids (full triangles). N is short for Naphthalene.

Deep seawater was sampled each year in the neighbourhood of the Rainbow hydrothermal field, in a zone unaffected by hydrothermal discharge, to be used as a control experiment for identification of contaminants from both deep seawater and sampling equipment. Figure 11 shows the characteristic organic signature, obtained using the current method, of deep seawater in the vicinity of the Rainbow hydrothermal field. The major peaks appeared to be phthalates and chlorinated compounds originating from the pipette tips. Minor peaks were normally of a totally different nature - mostly N, P and S bearing compounds - than the one detected in hydrothermal fluids. Only a few hydrocarbons and fatty acids were observed, nevertheless the number and variety of molecules belonging to each group of compounds was far lower than in the hydrothermal fluids (Table 1).

**Figure 12:**
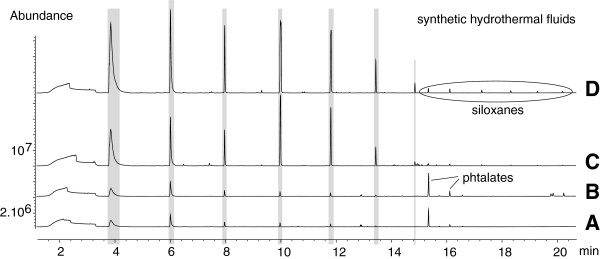
**TIC traces obtained after TD-GC-MS analysis of Twisters® stirred in synthetic hydrothermal solutions.A**: pH = 5.46, [Cl^-^] = 835 mM, [H_2_S] = 0.41 mM. **B**: pH = 3.13, [Cl^-^] = 305 mM. **C**: pH = 11.35, [Cl^-^] = 544 mM, [H_2_S] = 0.13 mM. **D**: pH = 11.86, [Cl^-^] = 5 mM, [H_2_S] = 3.3 mM. TICs were overlaid and are presented here with an offset for clarity.

Polymers may be altered and / or release compounds when exposed to the extreme pH, high H_2_S concentration and salinity of the hydrothermal fluids. PDMS stability and contamination issues with respect to pH, H_2_S concentration and variable salinity in experimental hydrothermal solutions have been studied elsewhere [13], however results are re-presented here. Twisters® were stirred in four solutions of various pH (3–12), salinity ([Cl^-^ = 5–835 mmol kg^-1^) and H_2_S concentration ([H_2_S] = 0–3.3 mmol kg^-1^) that mimicked different hydrothermal conditions encountered at MOR. The TICs obtained are shown in Figure 12. The above listed parameters did not significantly affect the PDMS phase. Siloxane abundances generally increased when Twisters® were stirred in basic solutions [34] (C and D on Figure 12). Phthalates of various origins (PDMS phase, pipette tips, plastic ware) were detected when extracting acidic solutions (A and B on Figure 12). However, regardless of their source they are clearly contaminants. Other complex molecules appeared to a lesser degree and were described by the authors as clearly distinctly different from hydrothermally derived compounds (i.e., compounds that are thought to be inherent to hydrothermal fluids samples as opposed to contaminants). They consist of common plasticizers or polymer industry-related compounds.

### Repeatability

In order to validate the repeatability of SBSE-TD-GC-MS for the analyses of organic compounds in hydrothermal fluids, a replicate experiment was carried out. Nine Twisters® were conditioned together (T91 to T98 and T100). T91 to T98 were stirred separately in aliquots of the same hydrothermal fluid sample (MOM-D4-Ti3), while T100 was kept as a dry blank reference (Table 2 and Figure 2). Seven of the stir bars were analysed sequentially, alternating with empty runs, on the same day and the last one was analysed on the following day. Toluene abundances differed among the samples (Figure 10). Strong abundance variations are commonly observed in TD-GC-MS (J. Guyomarch, personnal communication) Nevertheless, all TICs strictly superimposed (Figure 10), which shows an excellent qualitative repeatability of the entire method from sample preparation to analysis.

### Temporal sample stability

**Figure 13:**
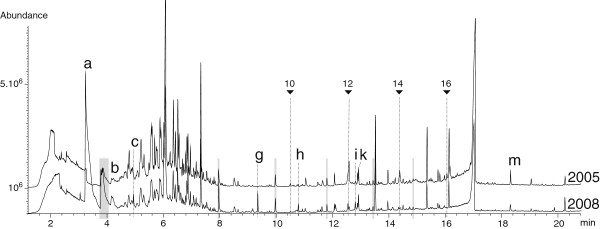
**TD-GC-MS TIC traces of SBSE extracts of 2 aliquots of the same fluid sample collected in 2005 from the Rainbow hydrothermal vent field.** Duplicate experiments are routinely carried out and here is a representative example of what was generally observed. T11 (refer to Table 2) was analysed in 2005 (top trace), whereas T10 (refer to Table 2) was analysed 3 years later (bottom trace). Examples of peaks that were not repetable are: (**a**) toluene; (**b**) benzene, chloro-; (**c**) oxime, methoxyphenyl; (**g**) decane, 1-chloro; (**h**) naphthalene, 2-chloro; (**i**) siloxane; (**k**) diethylphthalate; (**m**) benzene sulfonylbis, [4-chloro]. Black triangles refer to n-carboxylic acids with the number being the carbonated chain length.

T11 was analysed in 2005 just a few weeks after the end of the EXOMAR cruise during which samples were collected and extracted. A duplicate of the same sample extract (T10) was stored and analysed three years later. Figure 13 shows the TICs recorded after TD-GC-MS analysis of T11 and T10 (Table 2). The peaks attributed to the C_10:0–16:0_ carboxylic acids appeared smaller on the TIC obtained after 3 years of storage. At the present time and without the use of internal standards, we consider that the differences are due to a combination of common factors that affect the signal: aging of the machine and especially the electron multiplier, routine replacement of liner, as well as small differences in the vacuum level after maintenance of the apparatus. Some differences, such as the presence / absence of a peak, were also evident. They mostly affected the non-hydrothermally derived compounds, and a few examples of this are indicated in Figure 13. A general trend, observed not only in the TIC examples presented here, was that siloxane and chlorinated compound peaks seem to produce a much larger signal after a long storage period. Unfortunately, experimental restrictions prevented us from including more than one dry blank per batch of Twister® and one control experiment per cruise. Consequently, this interpretation will need to be tested in the future with the assistance of internal standards. Additional siloxanes commonly originate from other plastic ware such as septa. They were observed on TICs of empty runs which indicates an origin from the TD-GC-MS system itself (O-rings, septa…) Despite those few striking differences, TICs superimposed well and a detailed identification of each peak revealed the presence of the same hydrothermally derived organic compounds, i.e., n-alkanes, branched alkanes, cycloalkanes, aromatic hydrocarbons, PAHs and n-carboxylic acids in both samples [13]. Even though some variations in abundance may occur, no total loss of a compound or strong variations in terms of hydrothermally derived compounds were observed. All this infers that the recovered organic composition, using the current approach, is qualitatively preserved over a three-year storage period.

### Implication for the study of hydrothermal organic geochemistry

A wide variety of organic compounds have been recovered and analysed by SBSE-TD-GC-MS in numerous hydrothermal fluid samples. n-Alkanes, cycloalkanes, branched alkanes, BTEX and PAHs, as well as n-carboxylic acids have been identified and reported by Konn et al. [13] (Table 8). These occurrences are supported by field [4-8,14], experimental ( [49] and references therein) and theoretical observations [50]. Little is known of the overall geophysical and geochemical processes that control, on the one hand, the formation of organic compounds in hydrothermal systems and on the other hand, their distribution and dissolution in the fluid. First of all, mantle CO_2_ and living organisms are potential primary carbon sources to build up molecules. Secondly, several processes either abiogenic (catalytic reactions such as Fischer-Tropsch synthesis) [11,12,16], thermogenic [44] or biogenic (e.g. methanogens archaea) may be involved in the organic synthesis in hydrothermal systems. The extent to which each carbon source and process may contribute to the formation of organic compounds is unknown. Finally, seawater is thought to reach a supercritical state (Tc = 407°C, Pc = 298 bar) in seafloor hydrothermal systems and chemical reactions that take place under such conditions are largely uncharacterised [51]. The distribution and dissolution of organic compounds in hydrothermal fluids may be affected by the inorganic geochemistry and phase separation. The inorganic geochemistry of the Rainbow fluids is well documented. Several papers have presented evidence for the presence of a single fluid source fuelling all vents; concentrations of elements and gases have been stable for decades (e.g. [9,16]). However, the possibility of a link between the organic and the inorganic geochemistry of seafloor hydrothermal fluids has not been investigated. It is currently unknown whether the organic composition (type of compounds present, concentrations) varies with time or geographical position of hydrothermal systems. SBSE-TD-GC-MS analyses of hydrothermal fluid samples will be essential in understanding the geochemical processes controlling the organic geochemistry of hydrothermal fluids, as well as to investigate the influence of time and location on hydrothermal fluids organic geochemistry.

**Table 8:** List of major compounds proposed to be inherent of hydrothermal fluids and detected in in the Rainbow fluid samples

***Compounds***	***Molecular formula***	***Rt (min)***
toluene	C7H8	3.26*
ethylbenzene	C8H10	4.34*
p-xylene / m-xylene	C8H10	4.43*
cyclohexane, 1,?,? -trimethyl-	C9H18	4.48†
cyclohexane, 1,?,? -trimethyl-	C9H18	4.53†
cyclohexane, 1-methyl, ?-ethyl-	C9H18	4.65†
o-xylene	C8H10	4.71*
styrene	C8H8	4.71*
nonane	C9H20	4.76*
cyclohexane, 1-methyl, ?-ethyl-	C9H18	4.88†
cyclohexane, -propyl	C9H18	5.18†
branched alkane	C10H22	5.22†
cyclohexane, 1-ethyl, ?,?-dimethyl-	C10H20	5.33†
branched alkane	C10H22	5.51†
branched alkane	C10H22	5.59†
branched alkane	C10H22	5.70†
cyclohexane, 1-methyl, ?-propyl-	C10H20	5.89†
phenol	C6H6O	5.96†
decane	C10H22	6.05*
cyclohexane, 1-methyl, ?-propyl-	C10H22	6.18†
branched alkane	C13H28	6.25†
branched alkane	C13H28	6.33†
branched alkane	C11H24	6.38†
cyclohexane, butyl-	C10H20	6.52†
branched alkane	C11H24	6.57†
branched cyclohexane	?	6.70†
branched alkane	C11H24	6.80†
naphtalene, decahydro-, trans	C10H18	6.85†
branched alkane	C11H24	6.89†
branched alkane	C11H24	6.97†
branched cyclohexane	?	7.18†
undecane	C11H24	7.32*
cyclohexane, 1-methylbutyl-	C11H22	7.60†
branched alkane	C12H26	7.68†
cyclohexane, pentyl-	C11H22	7.82†
cyclopentane, hexyl-	C11H22	7.88†
branched alkane	C12H26	8.12†
branched alkane	C13H28	8.21†
octanoic acid	C8H16O2	8.24†
naphtalene	C10H8	8.52*
nonanoic acid	C9H18O2	9.43*
tridecane	C13H28	9.70*
naphthalene, ?-methyl-	C11H10	9.84†
n-decanoic acid	C10H20O2	10.52*
tetradecane	C14H30	10.79*
naphthalene, ?,?-dimethyl-	C12H12	11.21†
acenaphthene	C12H10	11.87*
dodecanoic acid	C12H24O2	12.58*
hexadecane	C16H34	12.81*
fluorene	C13H10	12.86*
heptadecane	C17H36	13.74*
tetradecanoic acid	C14H28O2	14.41*
octadecane	C18H38	14.62*
phenanthrene	C14H10	14.71*
anthracene	C14H10	14.81*
pentadecanoic acid	C15H30O2	15.23†
nonadecane	C19H40	15.47*
n-hexadecanoic acid	C16H32O2	16.12*
cyclic octaatomic sulfur	S8	17.10†
fluoranthene	C16H10	17.06*
pyrene	C16H10	17.47*
octadecanoic acid	C18H36O2	17.61*

Rt is the retention time obtained with the current analytical method. * this study. † [13].

**Figure 14:**
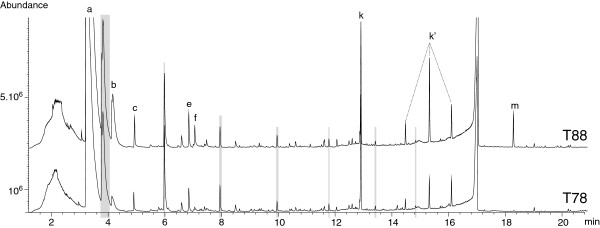
**These two TD-GC-MS TIC traces are a representative choice of the results obtained after Twister® extraction of the fluids from the Rainbow hydrothermal vent field collected in 2007 in the south-west area B, T88 (refer to Table**2**) (top) and in the north-east area C, T78 (refer to Table**2**) (bottom).** Examples of peaks that appeared in obvious different abundance are pointed out: (**a**) toluene; (**b**) benzene, chloro-; (**c**) oxime, methoxyphenyl; (**e**) octane, 1-chloro; (**f**) siloxane; (**k**) diethylphthalate; (k’) phthalatephthalates; (**m**) benzene sulfonylbis, [4-chloro].

**Figure 15:**
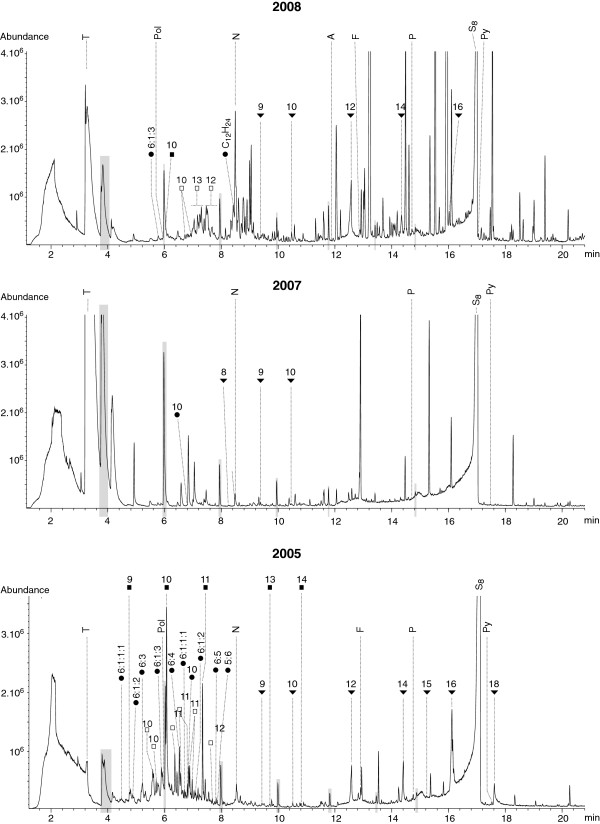
**These three TD-GC-MS TIC traces are a representative choice of the results obtained after Twister® extraction of the fluids from the Rainbow hydrothermal vent field in 2005 (bottom), 2007 (center) and 2008 (top).** Numbers stand for the carbonated chain length of n-alkanes (full squares), branched alkanes (empty squares) and n-carboxylic acids (full triangles). Cycloalkanes (full circles) are distinguished by the number of carbon in the cycle (first figure) and the number of carbon of the side chain(s) (following figure(s)). T, Pol, N, A, P and Py, and are short for toluene, phenol, naphthalene, acenaphthene, phenanthrene, and pyrene. Cyclooctaatomic sulfur (S_8_) was pointed because the peak was major. Highlighted in grey is the characteristic Twister’s signature. Monoaromatic hydrocarbons appeared too small and were not pointed on the TICs for sake of clarity.

We have demonstrated in the previous 4 sections that SBSE-TD-GC-MS is a method suitable for the qualitative analyses of a portion of the dissolved organic matter in small sample volumes of hydrothermal fluids. It is especially: (i) a method whose few contaminants can easily be distinguished, (ii) a repeatable method, and (iii) a method that enables storage of samples for at least three years. In addition, we obtained positive preliminary results from a successful comparative study dedicated to the Rainbow ultramafic-hosted hydrothermal field. Comparison of samples collected in different chimneys (T88 and T78; see Table 2, Figure 2) suggests a homogeneous organic geochemistry over the entire Rainbow field (Figure 14). In contrast, a time series study (sampling in 2005, 2007 and 2008) revealed strong variations over the years of the organic contents of the fluids from the Rainbow field (Figure 15, Table 8). These significant variations are real and meaningful. They are definitely not artifacts caused by the method of analysis or sample preparation technique used. They also are unlikely to be due to weekly or daily events such as tides because samples were collected at random times over a three to six-week period of time during cruises. These data show that this technology has a great potential for comparing the organic compositions of fluids originating from the same hydrothermal field (identical matrix). Therefore SBSE-TD-GC-MS analyses will be very useful in assessing intrafield variations (i.e., differences from one black smoker to another), in making interfield comparison, as well as carrying out time series studies.

Hydrothermal fluids most likely contain other organic molecules that cannot be recovered and / or detected using the current method. Firstly because of a molecule polarity issue [28], and secondly because some compounds may be undissolved (e.g., bound to salts or coordinated by metal ions). Therefore “organic composition” and “organic content” should only refer to the range of organic compounds that could be recovered and identified using our method, and likely represents a portion, consisting mainly of nonpolar compounds, of the total organic content of the hydrothermal fluids. All conclusions should only apply to that portion and in any case they should not be extrapolated to the total organic matter present in the hydrothermal fluids. However, SBSE has the versatility and the efficiency to greatly contribute to the understanding of organic geochemistry and geochemical processes of hydrothermal systems.

## Conclusions

This study has shown that SBSE-TD-GC-MS can be applied successfully for qualitative detection of a wide range of dissolved organic compounds in seafloor hydrothermal fluids. The organic content recovered using the current method likely represents a portion, with a predominance of nonpolar compounds, of the total dissolved organic matter present in the hydrothermal fluids. Precise identification and determination of the Rt of n-carboxylic acids, n-alkanes, BTEX and PAHs was achieved by comparison to synthetic standard mixtures. The analyses of eight replicates demonstrated the extremely good repeatability of the SBSE-TD-GC-MS method. Analyses of replicates of Twisters® stored for three years appeared to reproduce earlier results reliably, showing that SBSE is an excellent way of preserving the recovered organic signature of a sample. It is furthermore very suitable as a sample preparation technique to be used on board a research vessel and potentially in-situ.

The versatile and robust SBSE-TD-GC-MS technology allows comparative-qualitative studies provided the sample matrices are identical. In a case study, the recovered organic content of the fluids from the Rainbow Ultramafic-hosted hydrothermal system were compared in respect to the location and year of sampling. The same compounds were identified in fluids regardless of the sampling location. These preliminary results suggest a relative homogeneity in the dissolved organic content of fluids over the entire Rainbow field. Unlike, strong differences were observed among the years over the 2005–2008 time period.

The organic geochemistry of hydrothermal vents is highly relevant to issues of the origin of life on the early Earth and of the production of abiogenic hydrocarbons in these systems. In addition, organic compounds constitute carbon sources for microbial communities. The use of the SBSE-TD-GC-MS method for the study of hydrothermal organic geochemistry will contribute extensively to the understanding of the geochemical processes controlling the formation and distribution of the compounds as well as the interactions with rocks, minerals, metals and organisms.

## Competing interests

The authors declare that they have no competing interests.

## Authors’ contributions

CK carried out the SBSE analyses, interpreted the results and drafted the manuscript. JLC and JPD carried out sample collection, sample preparation for organic geochemistry and inorganic geochemistry analyses (pH, H_2_S, Cl^-^, etc.…). NGH participated in the design of the study and helped to draft the manuscript. All authors read and approved the final manuscript.
